# Anomalous Lobar Variations in Donor Lung: Challenges and Considerations in Lung Transplantation

**DOI:** 10.7759/cureus.101896

**Published:** 2026-01-20

**Authors:** Md Walid Akram Hussain, Md Tawseef Akram Hussain, Samuel Jacob

**Affiliations:** 1 Surgery, St. Joseph’s University Medical Center, Paterson, USA; 2 Surgery, Shaheed Suhrawardy Medical College and Hospital, Dhaka, BGD; 3 Cardiothoracic Surgery, Mayo Clinic, Jacksonville, USA

**Keywords:** anomalous lobe, cardiothoracic, lung, thoracic, transplant

## Abstract

Lung transplantation remains a life-saving procedure for patients with end-stage pulmonary disease. However, anatomical variations in the lung lobes, whether more or less than that normally described, present uncommon surgical and postoperative challenges in lung transplantation. This report presents a unique case of a four-lobed right lung and a three-lobed left lung encountered during organ retrieval, highlighting the complexities faced during transplantation as well as potential pitfalls during the surgery and subsequent postoperative follow-up care.

## Introduction

Lung transplantation remains an established modality for the management of patients with severe respiratory failure. The human right lung consists of three lobes (upper, middle, and lower), while the left lung consists of two lobes (upper and lower) with a lingula. This is due to the peculiarities of the development of the lungs during their intrauterine life. Around the fourth week of intrauterine life, the lung bud emerges as a ventral outpouching from the foregut and divides into two primary bronchial buds [1]. The right bud is further divided into three secondary bronchial buds, and the left bud into two, which further divide into tertiary bronchial buds to form the bronchopulmonary segments [2]. Normally, the spaces between these segments are obliterated, except at the principal bronchus, resulting in three lobes in the right lung and two in the left. The deep and complete fissures that develop are the oblique and horizontal fissures. However, the segments may not be completely obliterated, resulting in incompletely defined fissures or accessory fissures and even the absence of fissures altogether [3]. The presence of an extra lobe in the right lung or left lung, though rare, in some cases can affect both the donor lung selection process and surgical planning. In this case report, we discuss a unique case of a four-lobed right donor lung and a three-lobed left donor lung and explore how this may impact surgical and post-surgical complications of the graft.

## Case presentation

**Figure 1:**
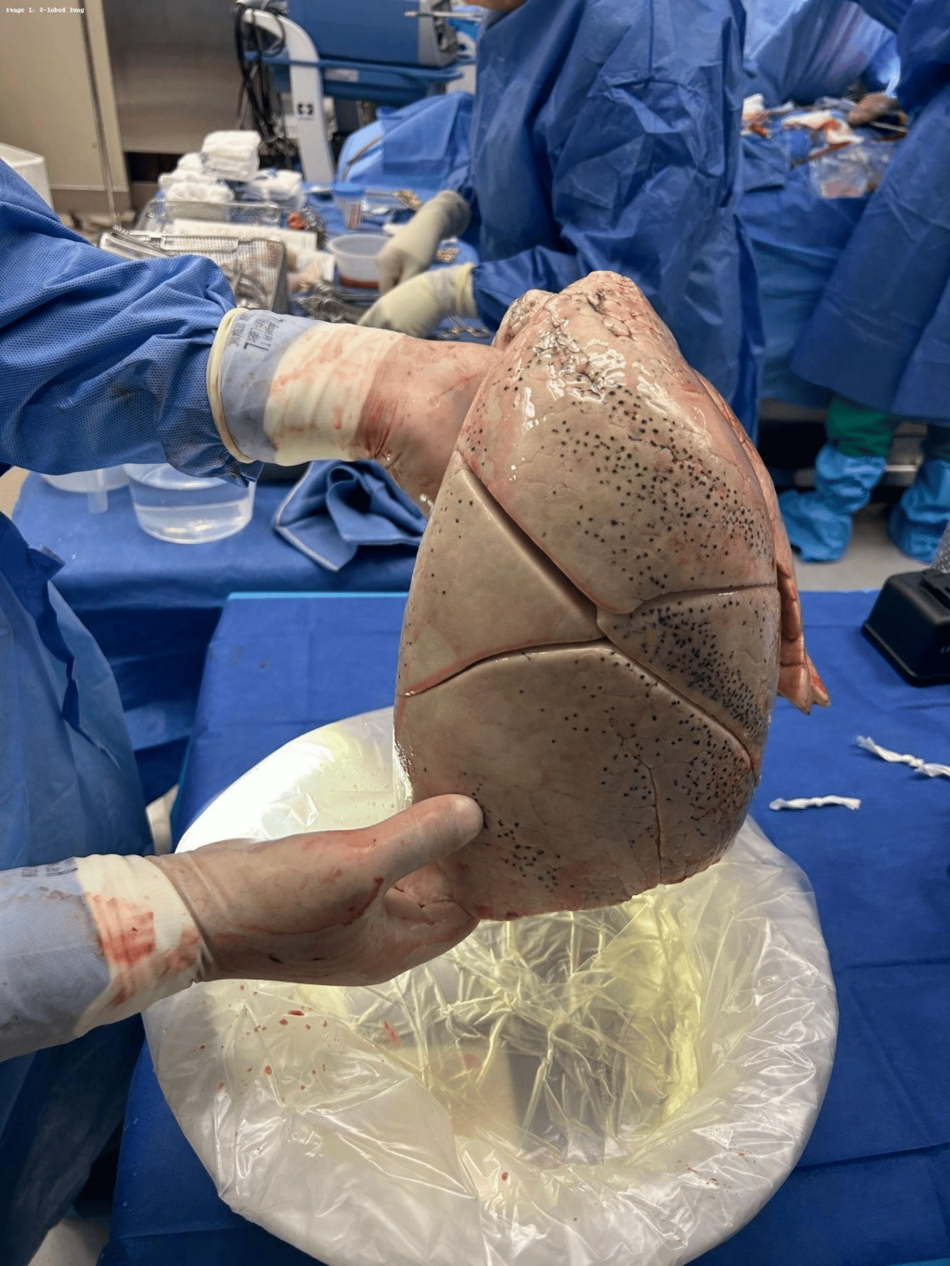
Four-lobed right lung. Intraoperative photograph of the explanted right lung demonstrating four distinct lobes, separated by well-formed fissures, representing a rare congenital anatomic variant. No preoperative imaging was available to identify this anomaly before organ retrieval.

**Figure 2:**
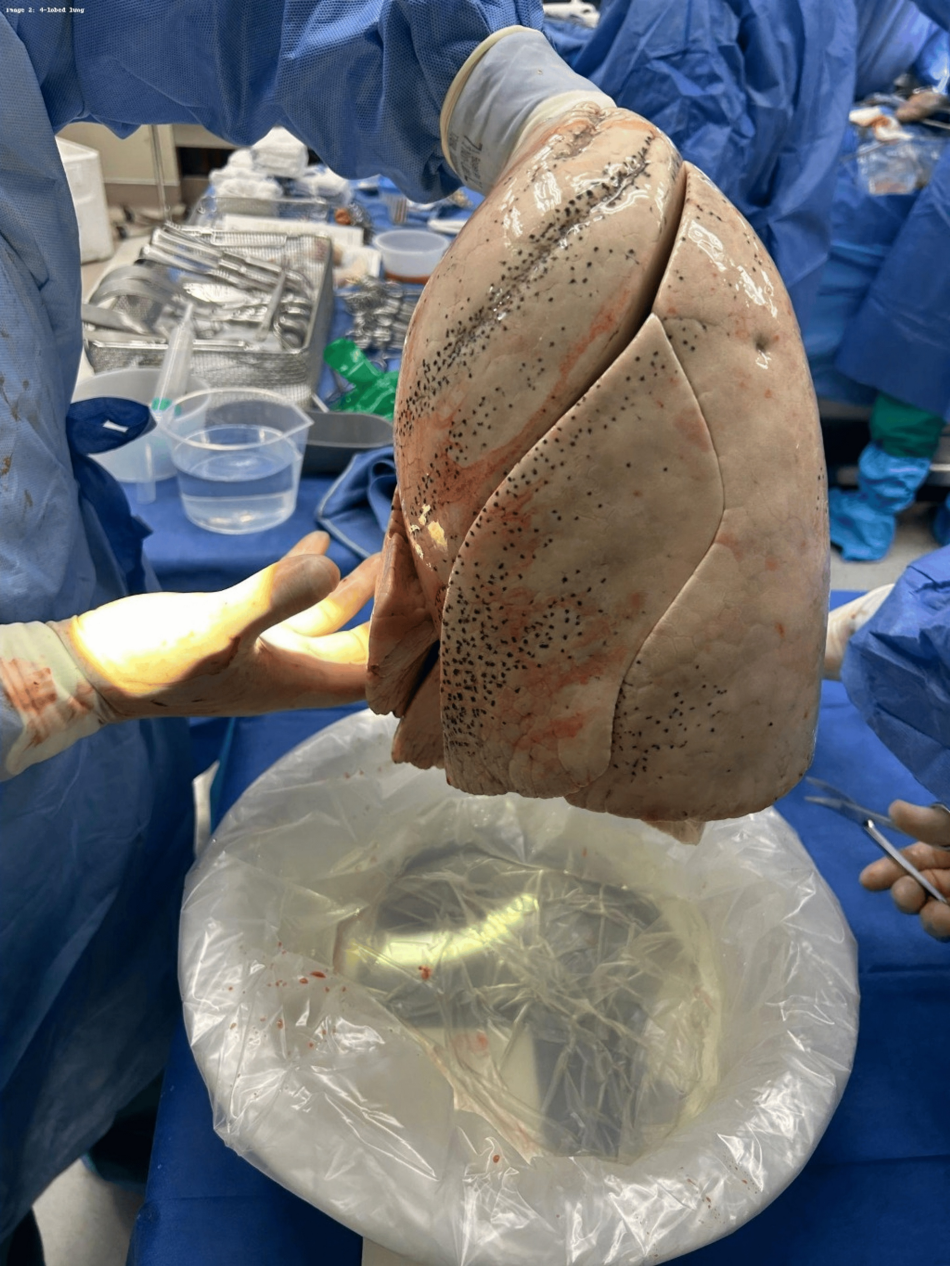
Three-lobed left lung. Intraoperative photograph of the explanted left lung demonstrating three distinct lobes with complete fissures, an uncommon anatomic variant as the left lung typically consists of two lobes.

A 39-year-old male presented to the emergency department after a self-inflicted gunshot wound to the head. On arrival to the trauma bay, he was found to have a Glasgow Coma Scale score of 3. The patient was emergently intubated for airway protection and underwent standard trauma resuscitation. Despite aggressive supportive care, neurological examination and subsequent evaluation were consistent with brain death, and the patient was pronounced brain dead. He was subsequently designated as a donor after brain death (DBD). No perioperative imaging was available before organ procurement. In the operating room, organ retrieval was performed using a standard procurement technique. Intraoperatively, the donor was noted to have an anatomic variation of the lungs, with a four-lobed right lung (Figure 1) and a three-lobed left lung (Figure 2), differing from typical pulmonary lobar anatomy.

Impact of anatomical variation on surgical planning

When a donor organ has additional lobes, meticulous planning must be done to account for the difference in size and anatomy. Some of the more important factors are discussed below.

Tailoring the Graft for the Recipient

Standard donor lungs typically have three lobes on the right and two on the left. Implantation of donor lungs with additional lobes into recipients with standard thoracic anatomy may lead to spatial and volumetric discrepancies. Strategies include downsizing donor lungs (lobar transplantation) [4,5] or expanding recipient pleural space intraoperatively [6,7]. In rare circumstances, anatomical ambiguity may contribute to logistical errors during organ allocation if not clearly communicated.

Surgical Approach

Due to the anatomic difference in donor lungs with an extra lobe, the standard transplantation techniques may have to be changed based on intraoperative findings. Intraoperative decisions may include resecting an extra lobe or preserving native lung segments for better post-transplant adaptation. A previous study described the anastomotic technique in lung transplantation involving additional lobes as being identical to that used in standard lung transplants [8].

Changes in Postoperative Follow-Up

It is important for the surgeon, transplant pulmonologist, and radiologist to be aware of this anatomical variation to avoid any confusion during the postoperative period. Potential sources of confusion include visualization of additional bronchi during bronchoscopy or misinterpretation of fluid within the accessory fissure as consolidation or pneumonia on imaging. There needs to be clear documentation in the patient’s operative note, as well as a bronchoscopy report of the anatomic variation, to avoid confusion by a physician who is unaware of the anatomical variation.

## Discussion

A lobar variation presents an additional level of complexity in lung transplantation. Anatomical matching between donors and recipients becomes more challenging when there is lobar variation between them. Surgeons must carefully plan for potential size mismatches and make intraoperative adjustments to ensure an optimal fit within the thoracic cavity, including downsizing the lung and/ or expanding the recipient pleural space.

Beyond the technical aspects of the procedure, postoperative management presents unique difficulties. Patients with extra lobes may experience altered ventilation-perfusion dynamics, which could lead to uneven lung aeration and higher rates of atelectasis [9].

Additionally, infections may pose a significant challenge in these cases. Surgical techniques compensating for variations in post-transplant bronchial anatomy due to the presence of additional donor lobes may cause altered bronchial anatomy. This can cause ineffective mucus clearance, predisposing patients to bacterial colonization and subsequent respiratory infections [10]. Moreover, an additional fissure may be misinterpreted as pneumonia or consolidation by radiologists. Hence, this can potentially make postoperative follow-up with imaging more challenging [8]. Consequently, postoperative radiologic assessment may be more challenging without prior awareness of anatomical variations. This includes explicit documentation of the anomaly as well as proper labeling to ensure no confusion arises.

Finally, the long-term outcomes of lung transplantation in patients with lobar anomalies remain an area requiring further study. To our knowledge, there have only been two previous cases of transplanting three-lobed donor lungs [8,11], and this is the first case of transplanting a four-lobed donor lung. The relative rarity of lung transplants involving additional lobes necessitates meticulous planning during surgery and afterward, and highlights the critical importance of clear documentation in operative and bronchoscopy reports.

## Conclusions

This report describes the first documented case of a four-lobed right donor lung and only the third reported case of a three-lobed left lung in the transplant setting. Recognition of such anatomical variations is essential, as they may significantly influence surgical planning, anastomotic technique, and postoperative management. Careful intraoperative assessment and clear documentation are critical to minimizing complications and optimizing graft outcomes.
